# Stereoselective Bacterial Metabolism of Antibiotics in Environmental Bacteria – A Novel Biochemical Workflow

**DOI:** 10.3389/fmicb.2021.562157

**Published:** 2021-04-16

**Authors:** Felicity C. T. Elder, Edward J. Feil, Ben Pascoe, Samuel K. Sheppard, Jason Snape, William H. Gaze, Barbara Kasprzyk-Hordern

**Affiliations:** ^1^Department of Chemistry, University of Bath, Bath, United Kingdom; ^2^The Milner Centre for Evolution, Department of Biology and Biochemistry, University of Bath, Bath, United Kingdom; ^3^AstraZeneca Global Sustainability, Mereside, Macclesfield, United Kingdom; ^4^European Centre for Environment and Human Health, University of Exeter Medical School, ESI, University of Exeter, Penryn, United Kingdom

**Keywords:** stereochemistry, antibiotic resistance, metabolism, environment, wastewater

## Abstract

Although molecular genetic approaches have greatly increased our understanding of the evolution and spread of antibiotic resistance genes, there are fewer studies on the dynamics of antibiotic – bacterial (A-B) interactions, especially with respect to stereochemistry. Addressing this knowledge gap requires an interdisciplinary synthesis, and the development of sensitive and selective analytical tools. Here we describe SAM (stereoselective antimicrobial metabolism) workflow, a novel interdisciplinary approach for assessing bacterial resistance mechanisms in the context of A-B interactions that utilise a combination of whole genome sequencing and mass spectrometry. Chloramphenicol was used to provide proof-of-concept to demonstrate the importance of stereoselective metabolism by resistant environmental bacteria. Our data shows that chloramphenicol can be stereoselectively transformed via microbial metabolism with *R,R-(-)-*CAP being subject to extensive metabolic transformation by an environmental bacterial strain. In contrast *S,S-(*+)*-*CAP is not metabolised by this bacterial strain, possibly due to the lack of previous exposure to this isomer in the absence of historical selective pressure to evolve metabolic capacity.

## Introduction

Antibiotics are a group of natural, semi-synthetic and synthetic pharmaceuticals that can inhibit the growth of, or kill, micro-organisms. Since their discovery 100 years ago antibiotics have revolutionised healthcare, animal husbandry and aquaculture to the point that they have now become indispensable within modern medicine and intensive food production. However, poor stewardship of antibiotics has contributed to their increased presence within the environment, where they are known to impact negatively on a range of organisms and ecosystems (Kumar et al., 2019). Once administered to patients or animals, antibiotics are often recalcitrant to metabolism within the treated host, and are readily excreted in an active form in human’s and animal’s urine (Hirsch et al., 1999).

Advances in hyphenated techniques, particularly liquid chromatography coupled with mass spectrometry, have facilitated the identification and quantification of antibiotics in a wide range of different environmental matrices, considerably improving our understanding of the fate of these compounds outside of the host (Carvalho and Santos, 2016), with multiple classes of antibiotics being reported in different environments worldwide (Kümmerer, 2003, 2009a, b, Michael et al., 2013; Larsson, 2014). This has led to a concerted effort in the form of the AMR (antimicrobial resistance) Industry Alliance from the pharmaceutical industry to understand and tackle how antibiotic production and discharge from production sites contributes to AMR. High concentrations and persistence of antibiotics (reaching and even exceeding μg/L levels) combined with known toxicity to aquatic organisms (González-Pleiter et al., 2013) have led to the inclusion of three macrolide antibiotics (erythromycin, clarithromycin and azithromycin) on the EU surface water watch list, as potential candidates for inclusion as priority pollutants in the EU Water Framework Directive (WFD) (Carvalho et al., 2008). As further evidence is generated concerning the consequences of antibiotic pollution of the environment with respect to the emergence of resistance, it is likely that further antibiotics will be added to the EU WFD watch list. With respect to the aquatic environment, a comprehensive understanding of the fate and effects of antibiotics within the water cycle is required to establish appropriate science-based guidelines and regulations. A critical component of this question, and one that has hitherto been almost completely overlooked, is the role of stereochemistry in determining the bioavailability and toxicity of individual antibiotics or their mixtures (Kasprzyk-Hordern, 2010).

Stereochemistry refers to the study of the relative spatial arrangements of atoms that form the structures of molecules; that is, their 3-dimensional shape. Chirality is a geometric property of molecules that are non-superimposable on their mirror image; stereoisomers that exhibit this property are called enantiomers. The chirality of a molecule is caused by the presence of one or more chiral elements, for example a chirality axis, plane or centre (e.g., tetrahedral carbon atom), and is indicated by the use of the *R and S* prefixes (Helmchen, 2016). Most antibiotics contain at least one chiral centre and one pair of enantiomers. Pure enantiomers of chiral compounds exhibit the same physicochemical properties, but due to different relative spatial arrangements of their atoms, their behaviour in biochemical processes can be strikingly different. Thalidomide is the most well-known example of where the two enantiomers have contrasting effects within the human body, with the *R-(-)-*enantiomer being harmless and having tranquilising properties while the *S-(*+)*-*enantiomer is a teratogen leading to birth defects when administered to pregnant women. In addition, the *R-(*+)*-*enantiomer is converted *in vivo* to the toxic *S-(*+)*-*form (Kasprzyk-Hordern, 2010), leading to its withdrawal for use as a morning sickness drug.

**FIGURE 1:**
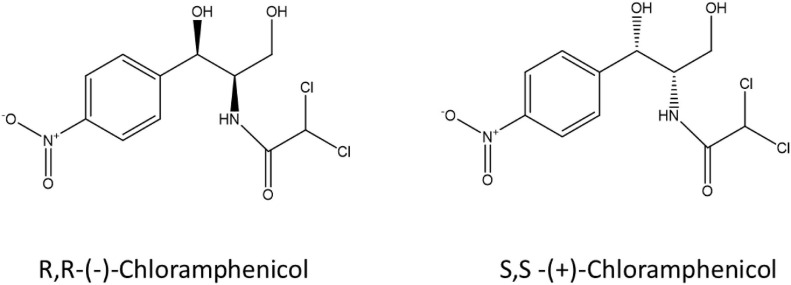
S,S-(+)-chloramphenicol and R,R-(-)-chloramphenicol.

Stereochemistry does not just play a role in human metabolism and toxicity but also in microbial metabolism of pharmaceuticals including fluoxetine, ephedrine and amphetamines (Chenevert et al., 1994; Bagnall et al., 2013; Evans et al., 2016; Andrés-Costa et al., 2017; Rice et al., 2018). Castrignanò et al. (2018) investigated the enantiomeric composition of ofloxacin in the aqueous environment, and verified the variable enantiomeric composition during wastewater treatment and in receiving waters showing enrichment of the *S-(-)-*enantiomer (Castrignanò et al., 2018). Enantioselective degradation of ofloxacin and levofloxacin by two strains of environmental bacteria has also been demonstrated (Maia et al., 2018) indicating a possible microbial aspect to enantiomeric enrichment in the environment. Despite published research on enantioselective microbial metabolism of pharmaceuticals, very little is known on the stereoselectivity of bacterial metabolism of antibiotics due to a lack of sensitive and selective analytical tools suited to complex environmental matrices at an enantiomeric level. Here we describe a laboratory workflow to test bacterial resistance mechanisms in the context of antibiotic-microbe interactions, the stereoselective antimicrobial metabolism (SAM) workflow. Two enantiomers of chloramphenicol: *S,S-(*+)*-*chloramphenicol and *R,R-(-)-*chloramphenicol (Figure 1) were used as a proof-of-concept to investigate stereoselective metabolism of an antibiotic by resistant environmental bacteria. Chloramphenicol was selected as a test antibiotic due to its physicochemical properties, including two chiral centres, as well as ongoing usage and resulting environmental presence. Chloramphenicol is the only over-the-counter antibiotic currently marketed in the United Kingdom. To our knowledge this is the first study that has explicitly addressed this question.

## Experimental Section

**FIGURE 2:**
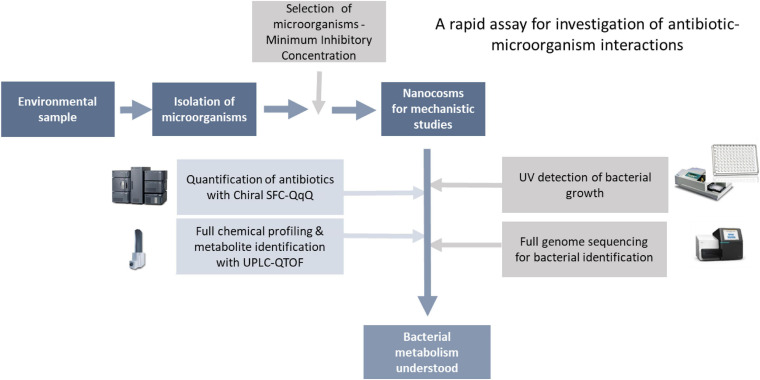
Analytical workflow.

As mentioned in the introduction, lack of tools to characterise antibiotic-bacteria interactions (A-B interactions) is a key hurdle in our understanding of the mechanisms behind antibiotic resistance (ABR). Here, we have developed a rapid biochemical assay enabling full characterisation of A-B interactions. As a proof-of-concept, we have focused on bacterial metabolism of chloramphenicol, where enzymatic transformation of the antibiotic is a key resistance mechanism. Although the assay can be used to study any A-B mechanism. The assay consists of two main steps: (1) isolation and characterisation of bacteria for testing, followed by (2) characterisation of antibiotic-microbe interactions in fully controlled nanocosms via profiling of microbial growth and antibiotic transformations (Figure 2).

### Materials

HPLC-grade acetonitrile (> 99.9%), HPLC-grade isopropanol (> 99.9%), HPLC-grade methanol (> 99.9%), HPLC-grade ethanol (> 99.9%), ammonium hydroxide, tryptic soy broth and *R,R-(-)-*chloramphenicol were supplied by Sigma-Aldrich (Gillingham, United Kingdom). *S,S-(*+)*-*chloramphenicol was supplied by TRC (Toronto, Canada) and by LGC Standards (Teddington, United Kingdom), D5-(±)-chloramphenicol were supplied by LGC Standards (Teddington, United Kingdom). *R,R-(-)-*chloramphenicol acetate was supplied by Santa Cruz Biotechnology (Dallas, TX, United States), Ultrapure water was obtained from a water purification system (MilliQ system United Kingdom). Brilliance UTI Chromogenic Agar was supplied in pre-poured plates by Oxoid (Basingstoke, United Kingdom). Qiagen QIAmp DNA mini prep kit (Qiagen) was used for DNA extraction and Imumina Nextra XT kit (Illumina) and V3-600 reagent cartridges (Illumina) were used for whole genome sequencing.

Stock solutions for each compound were prepared in acetonitrile and stored at −16°C. All glassware was deactivated with dimethylchlorosilane (5% DMDCS in toluene, Sigma-Aldrich) (Kasprzyk-Hordern et al., 2008).

### Methods

#### Isolation of Environmental Bacteria Strain

Raw and treated wastewater, as well as activated sludge samples were collected over two separate weeks in March and July from a wastewater treatment plant utilising activated sludge process. 10 μL wastewater or activated sludge were plated onto CLED agar plate containing 8 mg/L of *RR-(-)-*chloramphenicol and incubated overnight aerobically at 37°C. Selected colonies were initially identified through overnight growth on Brilliance UTI Chromogenic Agar (aerobically at 37°C) and stored at −80°C on Microbank beads for future analysis. One colony was taken forward based on MIC results for the development of a nanocosm assay aimed to investigate stereoselective transformation of antibiotics by environmental bacteria.

#### Minimum Inhibitory Concentration for *R,R-(-)-C*hloramphenicol, *S,S-(*+)*-*Chloramphenicol and Florfenicol

Bacterial strains were grown on CLED agar at 37°C aerobically overnight from Microbank bead. A McFarland 0.5 OD (600 nm) standard was made from the strain in sterile dH_2_O and 50 μL added to 4950 μL TSB to give approximately 1.5 × 10^6^ CFU per mL.

A stock solution of 256mg/L mg/L of each chloramphenicol enantiomer was made in sterile dH_2_O). MIC range of 126 - 0.25 mg/L was investigated using Strain 111 via the microbroth method in a 96 well plate. The 96 well plate was incubated aerobically at 37°C for 18 h in a Molecular Device SpectraMax3 with optical density readings at 600 nm being taken every minute. Florfenicol MICs were also performed as an indicator of the presence of the chloramphenicol acetlytransferase (Schwarz et al., 2004).

#### Nanocosm Assay

One hundred microlitre of 2 mg/L chloramphenicol (single enantiomer or racemic mix) in tryptic soy broth (TSB) was added to individual wells of a Thermofisher clear bottom 96 well microplate. For the abiotic assays, 100 μL of TSB was added to each abiotic well to give a final concentration of 1mg/L of total chloramphenicol (single or racemic). For the biotic assays a 0.5 MacFarland suspension of the strain being investigated was diluted 1:100 in TSB to give approximately 1.5 × 10^6^ CFU per mL. 100 μL of the TSB bacteria stock was added to the individual biotic wells to give a final concentration of 1 mg/L chloramphenicol (single or racemic). Bacteria growth was then measured by absorbance at 600 nm in a Molecular Devices SpectraMax3 plate reader every minute over a 24-h period. Samples were taken at four time points (T0h, T5h, T12h and T24h). Samples were diluted 1:10 in methanol with chloramphenicol D4 as an internal standard (100 ng/L), chilled overnight at 5°C and centrifuged for 5 mins at 13000 rpm. The supernatant was aspirated and placed into 500 μL plastic LC vials for analysis by SFC-QqQ and UPLC-QTOF as described below.

#### Chemical Analysis of Chloramphenicol Utilising Mass Spectrometry

##### Quantification of chloramphenicol with chiral SFC-QqQ

Analysis of chloramphenicol enantiomers was undertaken using Waters UPC^2^ supercritical flow chromatography coupled to Waters Xevo TQD equipped with electrospray ionization source (ESI) mode. Chromatographic separation of the chloramphenicol enantiomers was achieved using Waters Trefoil Amy1 column (2.5 μm, 2.1mm X 50mm) with the following mobile phases: CO_2_ (A) and 1:1:1 methanol:isopropanol:ethanol with 0.1% ammonium hydroxide (B). The gradient elution was as follows: 15% B (0-1min), 60% B (1–5 min), 60% B (5–7.5min), 15% B (7.5–7.8min), 15% B (7.8–9min). The flow rate was kept constant at 0.75 mL/min and column temperature set to 30°C. Injection volume was 3 μL.

The mass spectrometer was operated in the multiple reaction monitoring (MRM) mode. Molecular ions ([M-H]-) were selected in ESI-. MRM transitions and ESI parameters were obtained after direct infusion of each standard at a concentration of 100 ng/mL in the mass spectrometer. Optimised ESI parameters were as follows: capillary voltage 3.0 kV in ESI negative. The source temperature was 150°C and the desolvation temperature was 400°C. Nitrogen was used as nebulising and desolvation gas. The cone gas flow was 100 L/h and the desolvation gas flow was 550 L/h. Argon was used as the collision gas. Two MRM transitions (one for quantification and one for confirmation) were chosen for chloramphenicol. One MRM transition was selected for labelled internal standard. MRM transition for chloramphenicol were 320.8 > 151.8 (quantitative), 320.8 > 194.0 (qualitative). MRM transitions for chloramphenicol D5 was 325.9 > 157.0 (Table 1). The source and operating parameters were optimised for each transition as follows: CAP 320.8 > 151.8m/z, cone voltage (CV)/collision energy (CE): 27v/15v; CAP 320.8 > 194.0m/z, CV/CE: 35v/12v; CAP D5 325.9 > 157.0 m/z, CV/CE: 35v/20v. MassLynx 4.1 and TargetLynx (Waters, United Kingdom) were used to control the LCMS system and for data processing.

**TABLE 1:** Chiral SFC- QqQ MRM Transitions.

**Compound**	**Quantitative Transition**	**Qualitative Transition**
Chloramphenicol	320.8 > 151.8	320.9 > 194.0
Chloramphenicol D5	325.9 > 157.0	325.9 > 262.0

Eight-Point multi-component internal standard calibration curves were prepared in tryptic soy broth spiked before extraction and used for the quantification of chloramphenicol in tryptic soy broth samples. The calibration curve was prepared by calculating the ratios between the peak area of each substance and the peak area of the internal standard. Masslynx 4.1 software was used to analyse and process all data. Linearity and range of the analytical method were determined by dilution of a stock solution of (±)-chloramphenicol (12 ng/mL). Accuracy of the method was assessed as the percentage of deviation from the known amount of analyte added to the sample. Precision was evaluated as the relative standard deviation (RSD) of replicate measurements. Both intra- and inter-day reproducibility of the analytical method were assessed. Intra-day precision of the analytical method was evaluated over a short period of time under the same instrumental conditions. Nine determinations covered three concentrations for each enantiomer (100, 150 and 300 ng/mL) of tryptic soy broth spiked before extraction, three replicates each. Inter-day precision of the analytical method was verified by determinations that covered three concentrations (100, 150 and 300 ng/mL) of tryptic soy broth spiked before extraction, three replicates each analysed on three different days. Quantification and detection limits were determined using a signal-to-noise approach. Standard solutions diluted with sample diluents (tryptic soy broth) were used for method limit of detection and method limit of quantification (MDL and MQL, respectively). The enantiomeric fraction for *S,S-(*+)*-*CAP was calculated using the following equation: EF = (E1)/(E1 + E2) where E1 is the *(*+)-enantiomer and E2 is *(-)*-enantiomer concentration (calculated as ratio of analyte peak area to internal standard peak area).

##### Full chemical profiling and metabolite identification with UPLC-QTOF

Full chemical profiling and metabolite identification of chloramphenicol was performed by UPLC-QTOF using a previously published method (Lopardo et al., 2017). This method utilises a Dionex Ultimate 3000 HPLC (Thermo Fisher, United Kingdom, Ltd.) coupled to a Bruker Maxis HD Q-TOF (Bruker) equipped with an electrospray ionization source for all analyses. Nitrogen was used as nebulizing gas at a flow rate of 11 L/min at a temperature of 220°C at a pressure of 3 bar. The capillary voltage was set at 4500V and End Plate offset was set at 500 V. Analyses were performed in negative mode in both full scan mode (MS) and broadband CID acquisition mode (MS/MS). HyStar Bruker software was used to coordinate the LC-MS system. Waters ACQUITY UPLC BEH C18 column (50 mm × 2.1 mm, 1.7 μm) was used to achieve chromatographic separation of the metabolites and the following mobile phase composition: 1mM ammonium fluoride in water (A) and methanol (B). The flow rate was set at 0.4mL/min and the following gradient was used for the elution of compounds: 5% B (0–3 min), 60% B (3–4 min), 60% B (4–14 min), 98% B (14–17 min), 5% B (17.1–20 min). The source and operating parameters had been optimised as: capillary voltage, 4500 V; dry gas temperature, 220°C (N_2_); dry gas flow rate 12 L H^–1^ (N_2_); quadrupole collision energy. 4 eV; collision energy, 7 eV MS (full-scan analysis) and 20 eV MS/MS (bbCID mode). Nitrogen was used as the nebulizing, desolvation and collision gas. The method underwent full validation for the quantification of chloramphenicol and chloramphenicol 3-acetate with chloramphenicol D5 as internal standard.

#### DNA Extraction and Whole Genome Sequencing

Genomic DNA was extracted using a Qiagen QIAmp DNA mini prep kit (Qiagen) with lysis step. DNA was quantified, and quality assessed using the Nanodrop 2000 (ThermoFisher Scientific) Nextera XT libraries were prepared for sequencing using the benchtop Illumina MiSeq sequencer. Paired-end reads (2x300 bp) were generated using the v3-600 reagent cartridge. Using the online bioinformatics platform EDGE (v1.5.1), powered by MRC CLIMB^1^ (Connor et al., 2016; Li et al., 2017), sequenced reads were trimmed (unpaired reads smaller than 50 bp or with less than 5 times coverage were discarded) and assembled using SPAdes (Bankevich et al., 2012) (v3.9.1). Coding sequences were annotated with prokka (v1.11) and ShortBRED (Kaminski et al., 2015) was used to search assembled genomes for Antibiotic Resistance genes (CARD database) (Jia et al., 2017) and for virulence genes from (VFDB) (Chen et al., 2016).

## Results and Discussion

In order to test the hypothesis that environmental bacterial resistance mechanisms towards chloramphenicol can be stereoselective a laboratory workflow was developed which included the isolation and characterisation of environmental bacterial strains, their exposure to set concentrations of individual chloramphenicol enantiomers over a twenty four hour period and the chemical analysis of samples taken at set time points.

### Isolation and Initial MIC Screening of Environmental Bacteria

A wastewater treatment plant in the South West of England was sampled over two 7 day periods in the spring and summer. Chloramphenicol resistant microorganisms were isolated on CLED agar containing 4 mg/L of *R,R-(-)-*chloramphenicol. Initial identification of chloramphenicol resistant isolates was via Brilliance UTI Chromogenic agar coupled with susceptibility patterns to two chloramphenicol isomers, *R,R-(–)* and S,S-(+), and florfenicol. Strain 111 was given an initial identification as a member of the *Enterococcus* spp. using Brilliance UTI Chromogenic agar based on the ability of *Enterococcus* to metabolise β-glucosidase. Susceptibility testing was performed for *R,R-(-)-*chloramphenicol and *S,S-(*+)*-*chloramphenicol (Table 2). Florfenicol MICs were also performed as an indicator of the presence of the chloramphenicol acetlytransferase (Schwarz et al., 2004). Strain 111 showed resistance to *R,R-(-)-*chloramphenicol but was susceptible to florfenicol and therefore highly likely to contain the chloramphenicol acetyltransferase gene as a resistance mechanism toward chloramphenicol.

**TABLE 2:** Isolation and minimum inhibitory concentration for chloramphenicol of environmental bacterial strain from wastewater treatment plant.

**Strain**	**Location**	**MIC**		
		**R,R-(-) – CAP**	**S,S-(+) – CAP**	**Florfenicol**
111	WWTP influent	128mg/L	> 128mg/L	3.125mg/L

### Full Genome Sequencing

**FIGURE 3:**
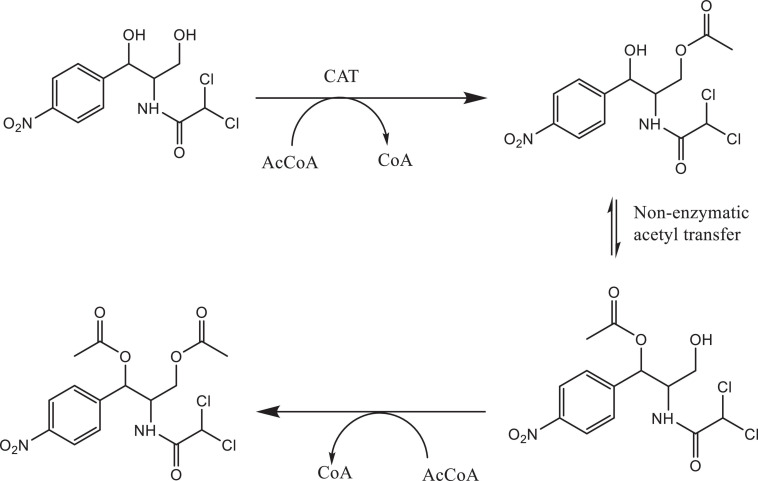
Acetylation of chloramphenicol by chloramphenicol acetyltransferase.

Bacterial species taxonomy and the mechanisms of chloramphenicol resistance was investigated in Strain 111 through genome sequencing. Strain 111 was confirmed as an *Enterococcus faecalis* and a number of enzymatic, target protection and efflux pumps identified as putative antibiotic resistance mechanisms, Table 3. Enzymatic resistance mechanisms include group A chloramphenicol acetyltransferase, lincosamide nucleotidyltransferase, aminoglycoside acetyltransferase, aminoglycoside nucleotytransferase, and aminoglycoside phosphotransferase. The strain also contains three efflux pumps, one specific (MLS antibiotics) and one multi-drug. Target protection resistance mechanisms includes tetracycline ribosomal protection protein and rRNA methylation. Chloramphenicol acetyltransferase is the only enzymatic resistance mechanism to chloramphenicol identified. This enzyme facilitates chloramphenicol resistance through the addition of acetyl groups to chloramphenicol (Figure 3) to form chloramphenicol acetate.

**TABLE 3:** Isolation, DNA extraction and whole genome sequencing of environmental bacterial strain 111 from wastewater treatment plant (raw wastewater).

**Strain**	**Nanodrop**		**Sequencing ID**	**Antibiotic Resistance**		
	**Nucleic acid [ng/μL]**	**A260/A280**		**Gene**	**Resistance Mechanism**	**Resistance**
111	114.3	2.021	Enterococcus faecalis	Cat8 isa ema tet-rpp ant erm aac aph	Chloramphenicol acetyltransferase ABC Efflux MFS Efflux Tetracycline ribosomal protection Aminoglycoside nucleotyltransferase rRNA methyltransferase aminoglycoside acetyltransferase aminoglycoside phosphotransferase	Chloramphenciol MLS* antibiotics Fluoroquinolone Tetracycline Aminoglycoside MLS* antibiotics Aminoglycoside Aminoglycoside

* - macrolide, lincosamide and streptogramin B.

### Stereoselective Transformation of Chloramphenicol in the Nanocosm Assay

**FIGURE 4:**
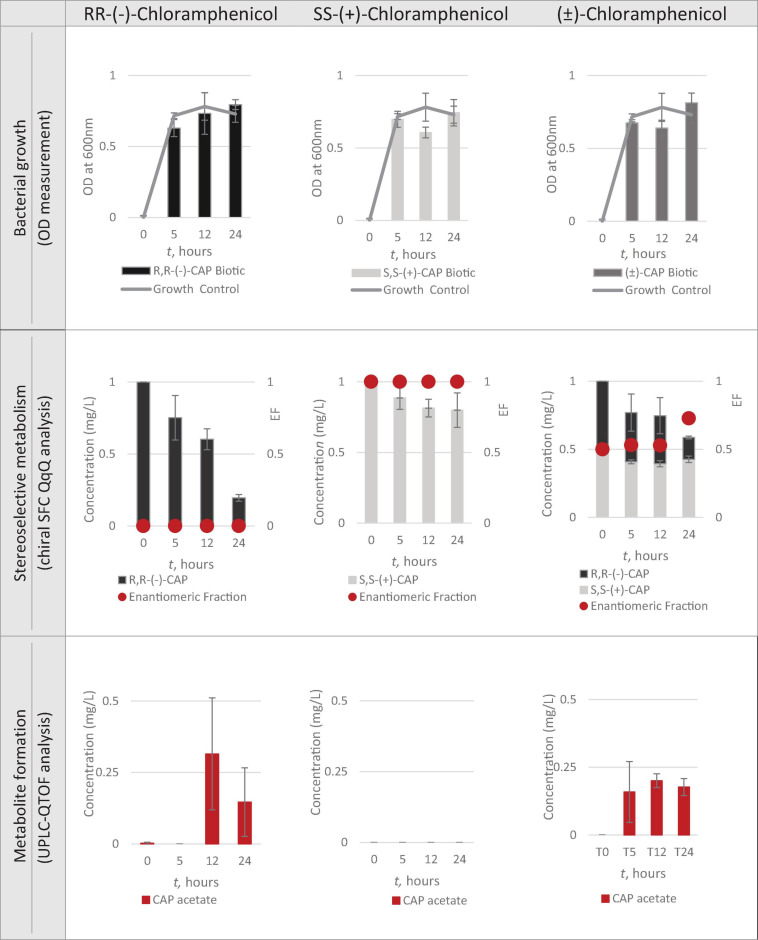
Nanocosm assay - biotic conditions (*n* = 3).

**FIGURE 5:**
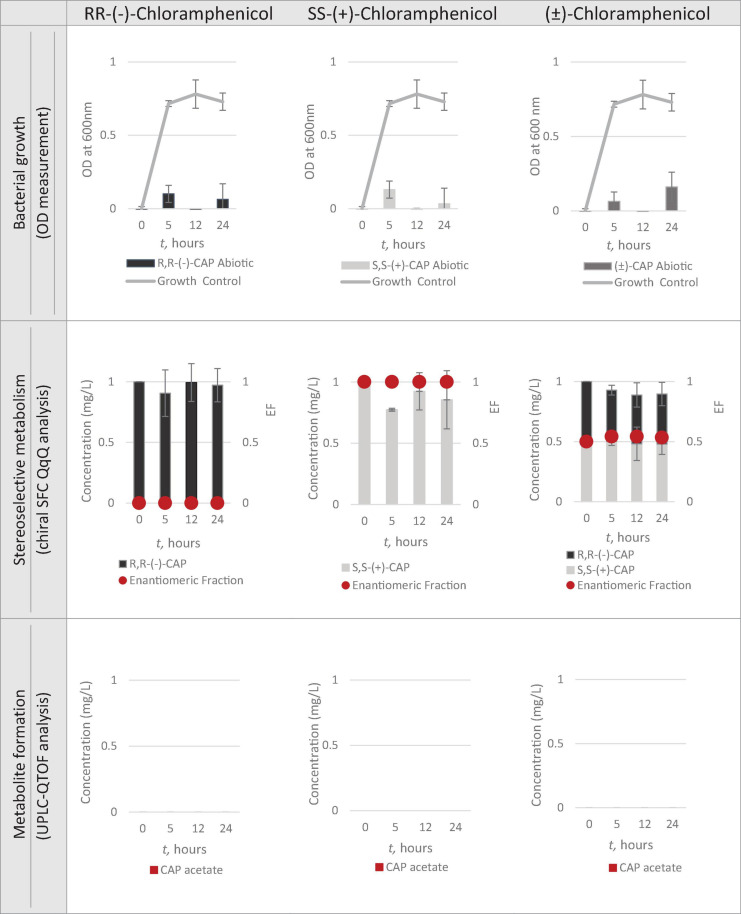
Nanocosm assay – abiotic conditions (*n* = 3).

Having identified Strain 111 as *Enterococcus faecalis* containing the CAT gene it was then used in the nanocosm experiment to test the hypothesis that CAT enzyme is stereoselective in its transformation of chloramphenicol. The conditions tested were the ability of Strain 111 to metabolise single enantiomer and racemic chloramphenicol at a 1mg/L aerobically at 37°C. Biological measurements were taken every minute through absorbance readings at 600nm to measure bacterial growth. Chemical samples were taken at time point 0h, 5h, 12h and 24h for chemical analysis with chiral SFC-MS/MS and HR-MS (Figure 4 for abiotic and Figure 5 for biotic). The sampling time points of T0, T5, T12, and T24 were selected in order to cover three of the four phases of bacterial growth lag, log and stationary phases.

#### Bacterial Growth

A total concentration of 1mg/L of chloramphenicol, a concentration lower than the MIC of both chloramphenicol enantiomers, was chosen for Strain 111 to ensure bacterial growth in the biotic nanocosms. Strain 111 was able to grow in the presence of *R,R-(-)-*chloramphenicol, *S,S-(*+)*-*chloramphenicol and *(*± *)-*chloramphenicol as indicated by the growth curves presented in Figure 4.

#### Quantification of Chloramphenicol With Chiral SFC-QqQ

##### Method performance

The new chiral SFC-QqQ method was fully validated before its application in nanocosm experiments (Table 4 and Supplementary Figure 1). Method validation revealed excellent intraday and inter-day accuracy and precision [intraday accuracy: 2.8% (R,R), 11.7% (S,S) and precision: 7.1% (R,R), 2.8% (S,S); inter-day accuracy: 17.8% (R,R) 4.0% (S,S) and precision: 3.0% (R,R) 4.9% (S,S)]. Ion ratios for two MRM transitions were 2.9 ± 5.3% (R,R) and 3.7 ± 11.9% (S,S). Very good linearity range [*R*^2^ = 0.995 (R,R) 0.994 (S,S)] was achieved within the following analyte concentration range: 5–600 μg/L. Method detection and quantification limits for chloramphenicol were as follows: MDL = 1 μg/L and MQL = 5 μg/L. Enantiomeric fraction was 0.39 ± 2.7%.

**TABLE 4:** Method performance for chiral SFC- QqQ.

**Chiral SFC- QqQ**	**Linearity range [μg/L]**	**R^2^**	**MDL [μg/L]**	**MQL [μg/L]**	**Accuracy [%]**		**Precision [%]**		**EF**	**Ion ratio [%]**
					**Intra-day**	**Inter-day**	**Intra-day**	**Inter-day**		
R,R-(-)-Chloramphenicol	5-600	0.995	1	5	2.8	17.8	7.1	3.0	0.39 ± 2.7	2.9 ± 5.3
S,S-(+)-Chloramphenicol	5-600	0.994	1	5	11.7	4.0	2.8	4.9		3.7 ± 11.9
										

##### Quantification of chloramphenicol isomers in nanocosm assays

Figures 4, 5 show chloramphenicol concentration change in nanocosm assays quantified with chiral SFC-QqQ method. As expected, in the abiotic samples (Figure 5) there is clearly no loss of either chloramphenicol enantiomer in the single enantiomer CAP nanocosms or racemic CAP nanocosms. In contrast, in the biotic nanocosms (Figure 4) there is clearly a loss of the *R,R-(-)-*chloramphenicol enantiomer in both the single and the racemic samples. The *S,S-(*+)*-*enantiomer stays relatively stable in concentration showing clearly the stereoselective transformation of chloramphenicol by the *Enterococcus faecalis* strain favouring *R,R-(-)-*enantiomer.

#### Full Chemical Profiling and Metabolite Identification With UPLC-QTOF

##### Method performance

The new UPLC-QTOF method was fully validated before its application in nanocosm experiments (Table 5). Method validation revealed excellent intraday and inter-day accuracy [intraday accuracy: 10% (CAP), 10% (CAP 3-acetate) and precision: 5% (CAP), 20% (CAP 3-acetate); interday accuracy: 20% (CAP) 5% (CAP 3-acetate) and precision: 5% (CAP) 5% (CAP 3-acetate). Very good linearity range (*R*^2^ = 0.998 (CAP) and 0.980 (CAP 3-acetate)] was achieved within the following analyte concentration range: 3–100 μg/L. Method detection and quantification limits for chloramphenicol were as follows: MDL = 1 μg/L and MQL = 3 μg/L. For chloramphenicol 3-acetate MDL = 1 μg/L and MQL = 3 μg/L.

**TABLE 5:** Method performance for UPLC – QTOF.

**UPLC- QTOF**	**Linearity range [μg/L]**	**R^2^**	**MDL [μg/L]**	**MQL [μg/L]**	**Accuracy [%]**		**Precision [%]**	
					**Intra-day**	**Inter-day**	**Intra-day**	**Inter-day**
Chloramphenicol	3-100	0.998	1	3	10	20	5	5
Chloramphenicol 3-acetate	3-100	0.980	1	3	10	5	20	5

##### Metabolic transformation of chloramphenicol in nanocosm experiments

In order to test the hypothesis that the stereoselective transformation of chloramphenicol by Strain 111 is metabolic in nature, nanocosm samples were analysed with UPLC-QTOF. The results presented in Figure 4 clearly indicate formation of the bacterial metabolite chloramphenicol 3-acetate where the bacterial strain has been exposed to the single enantiomer *R,R-(−)-*chloramphenicol in contrast to the abiotic samples shown in Figure 5. An increase in concentration of chloramphenicol acetate is proportional to the decrease in the concentration of *RR-(−)-*chloramphenicol. Where *E. faecalis* was exposed only to the *S,S-(*+)*-*chloramphenicol there was no formation of the metabolite chloramphenicol 3-acetate and no decrease in chloramphenicol concentration. As a result of exposure of the strain to *(*± *)-*chloramphenicol, chloramphenicol 3-acetate was also observed, though not to the same concentration levels as when exposed to single *R,R-(−)-*enantiomer; similarly, the concentration of chloramphenicol did not decrease to the same extent. This clearly indicates that the metabolism of chloramphenicol via the chloramphenicol acetyltransferase enzyme occurs in studied nanocosms and is highly stereoselective.

## Conclusion

Here, an interdisciplinary workflow for the investigation of chemical transformation and microbial processes has been developed in order to enable the investigation of antibiotic resistance and associated degradative mechanisms in environmental bacteria. The newly developed assay was used to test the hypothesis that enzymatic bacterial resistance can be stereoselective in nature. Chloramphenicol was used as a proof-of-concept antibiotic. The stereoselective antimicrobial metabolism (SAM) workflow provided, for the first time, clear evidence supporting our hypothesis that chloramphenicol can be stereoselectively transformed via microbial catabolic processes.

*R,R-(−)-*chloramphenicol is subject to extensive metabolic transformation by a chloramphenicol acetyltransferase enzyme, which appears to be capable of only metabolising this particular isomer. This result is readily explained firstly by higher exposure levels of environmental bacteria to this particular isomer and the fact that *R,R-(−)-*chloramphenicol is the naturally produced isomer meaning there has potentially been co-evolution of resistance mechanisms. *S,S-(*+)*-*chloramphenicol on the other hand is not metabolised by the bacteria we tested, and this is consistent with a lack of previous exposure to this form of chloramphenicol.

Current fragmented knowledge of ABR and our limited understanding of the chemical processes underlying the fate of antibiotics within the environment in the context of antibiotic-bacterial interactions jeopardises progress in this area. The stereochemistry of antibiotics and the role it plays in the fate of antibiotics in the environment including in the selection and persistence of ABR is one of the most critical but overlooked phenomenon in our battle against ABR. The lack of acknowledgment of stereochemistry in environmental risk assessment and establishment of PNEC or MIC levels disregarding non-racemic occurrence of chiral antibiotics in the environment, could potentially lead to inappropriate environmental standard safety levels and consequently sub-optimal management of ABR hotspots in the environment.

## Data Availability Statement

The datasets presented in this study can be found in an online repository: https://www.ncbi.nlm.nih.gov/bioproject/PRJNA639697.

## Author Contributions

FE: conceptualisation, writing, data collection, and interpretation. EF, JS and WG: conceptualisation and supervision. BP and SS: full genome sequencing. BK-H: conceptualisation, writing, and supervision. All authors contributed to the article and approved the submitted version.

## Conflict of Interest

JS was employed by the company Astra Zeneca. The remaining authors declare that the research was conducted in the absence of any commercial or financial relationships that could be construed as a potential conflict of interest. The authors declare that this study received funding from Astra Zeneca. The funder (JS) had the following involvement with the study: conceptualisation of the study and supervision.

## Footnotes

1 https://edgebioinformatics.org
