# Supplementary material for: Stereoselective Bacterial Metabolism of Antibiotics in Environmental Bacteria – A Novel Biochemical Workflow
Source: Front Microbiol. 2021 Apr 16;12:562157. doi: 10.3389/fmicb.2021.562157 (PMC8086513; doi:10.3389/fmicb.2021.562157)
Supplement: Supplementary file 1 [file Data_Sheet_1.pdf]

Supplementary material

Stereoselective bacterial metabolism of antibiotics in environmental bacteria – a novel biochemical workflow

Felicity C T Elder[1], Edward J Feil[2], Ben Pascoe[2], Samuel K Sheppard[2] Jason Snape[3], William H Gaze[4], Barbara Kasprzyk-Hordern[11]

[1] *Department of Chemistry, University of Bath, BA27AY, Bath, United Kingdom*
[2] *The Milner Centre for Evolution, Department of Biology and Biochemistry, University of Bath, BA27AY, Bath, UK*
[3]*AstraZeneca Global Sustainability, Mereside, Macclesfield, SK10, 4TG, UK*
[4]*European Centre for Environment and Human Health, University of Exeter Medical School, ESI, University of Exeter, Penryn Campus, Penryn, TR10 9FE, UK*

Fig S1. Chiral separation of (±)-chloramphenicol and (±)-chloramphenicol D5

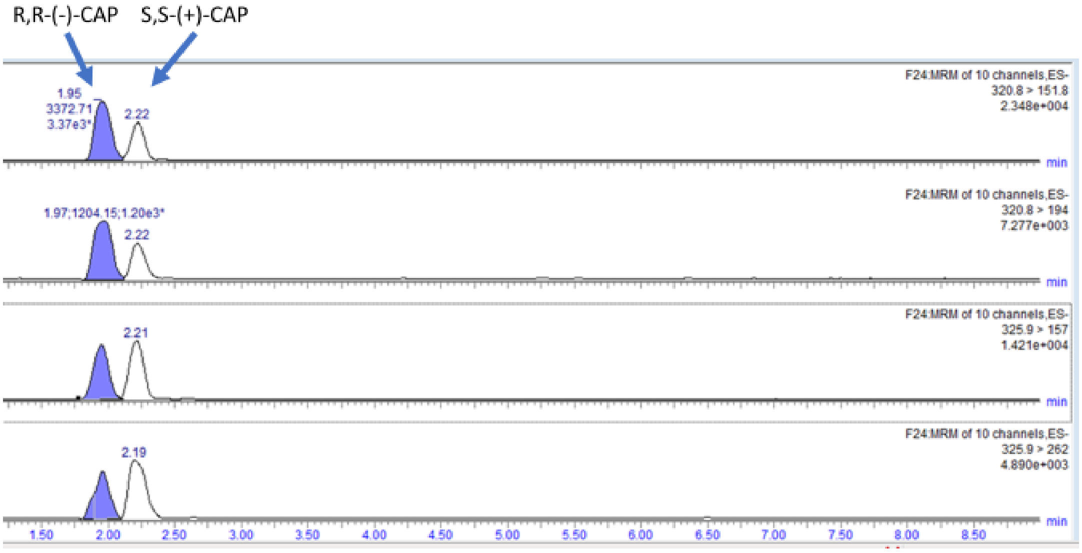

Fig S2. UPLC-QTOF separation of chloramphenicol and chloramphenicol acetate

[1] Corresponding author: E-mail: B.Kasprzyk-Hordern@bath.ac.uk; Fax: +44(0) 1225 386231; Tel: +44 (0) 1225 385013

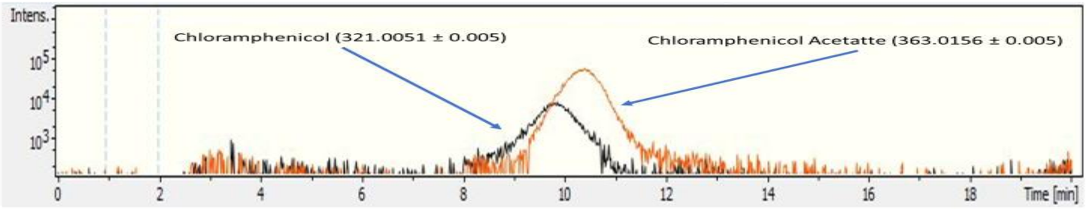
Intens.
10^5
10^4
10^3
Chloramphenicol (321.0051 ± 0.005)
Chloramphenicol Acetatte (363.0156 ± 0.005)
0
2
4
6
8
10
12
14
16
18
Time [min]
